# Pandemic (H1N1) 2009 influenza A in Universiti Kebangsaan Malaysia Medical Centre in 2009-2010: laboratory diagnostic test methods and lessons learned

**DOI:** 10.1186/1753-6561-5-S6-P87

**Published:** 2011-06-29

**Authors:** Z Zainol Rashid, KK Wong, H Mahbob, I Isahak

**Affiliations:** 1 Dept of Medical Microbiology & Immunology, Faculty of Medicine,Universiti Kebangsaan Malaysia, Kuala Lumpur, Malaysia; 2 Faculty of Medicine & Health Sciences, Universiti Sains Islam Malaysia, Kuala Lumpur, Malaysia

## Introduction / objectives

Pandemic (H1N1) 2009 Influenza A virus has moved into the post-pandemic period since August 2010. Various laboratory diagnostic methods are available to assist patient management. The study aimed to assess influenza trends in this centre during and post-pandemic and the performance of rapid influenza antigen diagnostic tests (RIDT).

## Methods

Data from August 2009 to December 2010 were collected. In high-risk patients and those with moderate to severe influenza-like illness (ILI), diagnosis of influenza A and pandemic (H1N1) influenza were confirmed by real-time reverse-transcription polymerase chain reaction (rRT-PCR) by Roche LightCycler® 2.0 system. Seven RIDTs were used and results were compared with rRT-PCR to assess performance.

## Results

In total, 733 respiratory specimens were tested from August to December 2009, where 165 (22.5%) were H1N1 influenza while 170 (23.2%) were seasonal influenza A. In 2010, out of 871 specimens tested, only 46 (5.3%) were H1N1 influenza while 97 (11.1%) were seasonal influenza A. RIDTs done on 1003 out of 1604 specimens showed low or variable sensitivities and negative predictive values, and generally high specificities and positive predictive values.

## Conclusion

In post-pandemic period, H1N1 influenza continues to circulate at low levels. A proportion of patients with ILI may require further diagnostic tests for other respiratory pathogens since only a small percentage were confirmed as influenza by rRT-PCR. RIDTs have low sensitivities and negative results should be confirmed with more sensitive methods. Based on the limitations and advantages of the tests, clinical and epidemiologic data is integral in patient assessment and interpretation of results.

## Disclosure of interest

None declared.

